# Emerging Applications of Radiomics in Neurological Disorders: A Review

**DOI:** 10.7759/cureus.20080

**Published:** 2021-12-01

**Authors:** Houman Sotoudeh, Amir Hossein Sarrami, Glenn H Roberson, Omid Shafaat, Zahra Sadaatpour, Ali Rezaei, Gagandeep Choudhary, Aparna Singhal, Ehsan Sotoudeh, Manoj Tanwar

**Affiliations:** 1 Radiology, University of Alabama at Birmingham, Birmingham, USA; 2 Radiology, University of Semnan, Semnan, IRN; 3 The Russell H. Morgan Department of Radiology and Radiological Science, Johns Hopkins University School of Medicine, Baltimore, USA; 4 Radiology, University of Alabama at Birmingham School of Medicine, Birmingham, USA; 5 Radiology, Oregon Health & Science University, Portland, USA; 6 Surgery, Iranian Hospital in Dubai, Dubai, ARE

**Keywords:** artificial intelligence, demyelinating disorders, stroke, neurology, radiomics

## Abstract

Radiomics has achieved significant momentum in radiology research and can reveal image information invisible to radiologists’ eyes. Radiomics first evolved for oncologic imaging. Oncologic applications (histopathology, tumor grading, gene mutation analysis, patient survival, and treatment response prediction) of radiomics are widespread. However, it is not limited to oncologic analysis, and any digital medical images can benefit from radiomics analysis. This article reviews the current literature on radiomics in non-oncologic, neurological disorders including ischemic strokes, hemorrhagic stroke, cerebral aneurysms, and demyelinating disorders.

## Introduction and background

While no new imaging modalities have been invented within the last few decades, technological advances in image acquisition and analysis have greatly improved the diagnostic utility of radiologic imaging. In recent times, the emergence of new segmentation software packages (e.g., neural networks) has led to the rise of quantitative image analysis in radiology [1]. A more sophisticated concept is converting digital medical images into mineable high-dimensional data, a process known as radiomics [2]. Traditionally, radiologists use a limited number of imaging features for diagnosis such as lesion size, density, lesion border, and enhancement. In contrast, radiomics can easily use many more quantitative features to predict and capture specific medical information. Radiomics relies on identifying potentially non-obvious imaging “features.” Here, “features” refers to the mathematical characteristics of the image.

Basics of radiomics

Radiomics is the high-throughput extraction of large amounts of quantifiable information from a region of interest (ROI) in digital medical images [3]. By converting medical images into hundreds or thousands of quantitative imaging features via data characterization algorithms, radiomics can extract information invisible to the human eye [4]. The extracted features can then be evaluated to make predictions, and the most predictive features are selected. Then, predictive models are built on top of these selected features to make predictive decisions regarding various medical conditions.

A radiomics pipeline starts with image processing. In image processing, different medical images from various centers and vendors are normalized and harmonized to neutralize the effect of different scanners and reconstruction techniques. After normalization of the images, the area of interest (lesions) is segmented. For segmentation, two-dimensional or three-dimensional images can be used; moreover, segmentation can be manual (done by an expert radiologist), semi-automated, or fully automated. The segmented area is then analyzed using a computational method, and hundreds and thousands of different quantitative features are extracted from the segmented regions. These quantitative features are essentially the mathematical relationship between different pixels and voxels of the segmented area. The most commonly used radiomics features are intensity (histogram) features, shape features, and texture features [5]. In addition, different types of filters (wavelet and Gaussian filters) can be applied to the images before feature extraction, which can multiply the number of extracted features many times. The extracted features are then used to train different machine learning models for different predictions such as tumor grade, histopathologic diagnosis, gene mutations, treatment response, patient survival, complications, and many more clinically relevant outcomes [6]. However, using hundreds and thousands of features to train the predictive learning models is not ideal. It is well-known that if a predictive model is trained with “too many” features, the subsequent trained model will work well on the training dataset but poorly in the real world (also referred to as overfitting in machine learning). To avoid this challenge, only the most predictive features must be utilized for model training, and the rest of them should be discarded. This act is referred to as “feature selection.” There are many different approaches to finding the most predictive features, and most of them are mathematical-based platforms. In radiomics, one of the most commonly used feature selection techniques is the least absolute shrinkage and selection operator (LASSO) [6].

**Figure 1:**
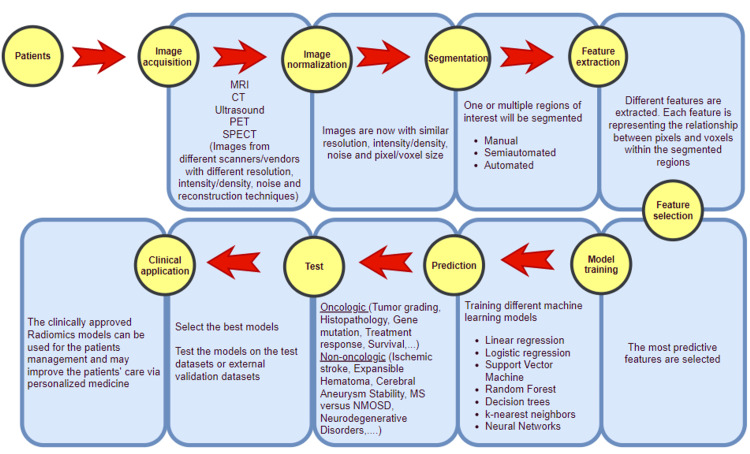
The radiomics pipeline. MRI: magnetic resonance imaging; CT: computed tomography; PET: positron emission tomography; SPECT: single-photon emission computed tomography; MS: multiple sclerosis; NMOSD: neuromyelitis optica spectrum disorder

Different machine learning models (neural networks, random forest, decision trees, support vector machine [SVM], etc.) are then trained to utilize the selected features to predict one element of a specific clinical question. Each trained model is then tested either on a test dataset, or an external dataset, or via cross-validation. Performance of models is usually reported by accuracy, sensitivity, specificity, and area under the curve (AUC). AUC is a standard metric for performance measurement, especially implemented to test the performance of various machine learning approaches. AUC ranges from 0 to 1, with values closer to 1 suggesting a better predictive model. The most robust predictive model is then reported. It is very common to summate different models to create a higher-performing model (ensemble techniques) [7]. A flowchart depicting the various steps of the radiomics pipeline has been summarized in Figure 1.

## Review

Applications in neurological disorders

The first applications of radiomics in imaging were in the field of oncology. This was also due to the support from various genomic projects and abundant biomolecular research data, which motivated researchers to devote their efforts to oncologic applications [8]. So far, the most common application of radiomics regarding oncologic imaging is the prediction of histopathology, tumor grading, genetic mutations, prediction of treatment response, the chance of recurrence, and patient survival. However, the application of radiomics is not limited to oncologic imaging and can essentially be extended to any clinical condition [5-7]. Taking inspiration from successes in the field of oncologic imaging, researchers have started to apply these techniques in non-oncologic diseases. The non-oncologic applications of radiomics in neurology are now emerging. The most common neurologic conditions (ischemic strokes, hemorrhagic stroke, cerebral aneurysms, and demyelinating disorders) are the most evaluated neurologic diseases by radiomics.

In this paper, we review the most common applications of radiomics in the field of neurology. After a comprehensive search within the PubMed database, the related published studies in English were selected. After reviewing the articles, only studies with a complete radiomics pipeline (clear information about the patient population, segmentation techniques, radiomics software, number of total features, feature selection model, number of selected features, machine learning models, and model performance) were selected for this review. Applications regarding cerebral infarction and hemorrhage, cerebral aneurysms, and demyelination disorders are included. Radiomic applications for neurodegenerative and psychiatric disorders are beyond the scope of this review.

Ischemic stroke

Stroke is the leading cause of disability and the fifth most common cause of death in the United States, with the majority (80%) of cases attributed to an ischemic etiology. Detection of ischemic stroke in the early hyperacute (0-6 hours) and late hyperacute stage (6-24 hours) is critical in the selection of patients for intravenous or mechanical thrombectomy [9]. Predicting the outcome of the intervention, including accurate identification of the Alberta Stroke Program Early CT Score (ASPECTS), penumbra tissue, successful revascularization, and risk of hemorrhagic transformation after treatment, are crucial in the long-term outcome and prognosis [10,11].

Infarction Detection

Time is a critical factor for stroke management. Early intravenous (IV) tissue plasminogen activator injection and/or thrombectomy are required to prevent cell death. Comprehensive stroke programs expedite the timely transportation of the patient from the Emergency Department (ED) to the computed tomography (CT) scanner. Patients with stroke symptoms undergo brain CT as per the modern stroke guidelines. Brain CT during this hyperacute phase usually has no appreciable finding for radiologists. However, preliminary studies have indicated that the difference between the normal and infarcted brain tissue can be detected by radiomics techniques and texture analysis [12]. In another study on 139 non-contrast brain CT scans of hyperacute brain infarction (within eight hours of infarction), texture analysis differentiated between normal and infarcted brain tissue with an AUC of 0.82. Interestingly, radiomics for hyperacute infarct was not time-dependent, and performance in two hours after symptom onset was the same as eight hours after symptom onset. Further, radiomics performance did not depend on the infarction size [13].

Thrombosis Characterization

Appropriate selection of patients for mechanical thrombectomy is a major clinical dilemma, especially in patients presenting six hours after symptom onset and in cases presenting with wake-up stroke when the time of symptom onset cannot be ascertained. Recent stroke trials have shown increased indications to offer mechanical thrombectomy to these patients. In this context, the RAPID automated CT perfusion platform (iSchemaView, Inc., Menlo Park, CA, USA) is widely used and helps physicians design the treatment plan by providing a relatively accurate estimation of the volume of infarction and ischemic penumbra, especially for patients presenting late [14]. Radiomics may play a role in patient selection for ischemic stroke treatment. The composition of intraluminal thrombus is an active area of scientific research as it can have a direct bearing on the first-attempt recanalization and the number of passages required for a successful recanalization (thrombolysis in cerebral infarction [TICI] grading system of 2c and 3 on cerebral angiography). It has been shown that segmentation of the vascular thrombosis on non-contrast brain CT with subsequent feature extraction and feature selection can predict the difficulty of the endovascular and thrombolytic treatment in patients with ischemic stroke. In a previous study, nine features extracted from the intraluminal thrombus were predictive of the successful recanalization with an AUC of 0.88 [15]. In another study, internal carotid artery/M1 thromboses in 67 patients were analyzed using radiomics. A total of 326 features were extracted from thrombosis on both non-contrast CT and CT angiography (CTA). SVM, built on top of the most predictive features, achieved an AUC of 0.85 to predict recanalization after IV alteplase treatment [16].

Identification of High-Risk Carotid Plaque

It is well-known that not all carotid atherosclerotic plaques causing less than 50% stenosis are associated with an increased risk of brain infarction. Traditionally, the presence of intraplaque bleeding and lipid-rich necrotic core are considered indicators of unstable plaques. In one study, radiomics was used to extract multiple features from the carotid plaques on T1, T2, and magnetic resonance angiography (MRA) (time of flight). The most predictive features were selected by LASSO and used to predict the symptomatic atherosclerotic plaques in carotids. In the study, traditional techniques utilized by radiologists had an AUC of 0.80, while the radiomics approach achieved an AUC of 0.98 for this task [17].

Prediction of Malignant Middle Cerebral Artery Infarction

Malignant middle cerebral artery (MCA) infarction is one of the most severe complications of cerebral infarction. It presents with rapid deterioration of neurologic status after large MCA infarction, mainly because of severe edema and mass effect with an associated mortality of approximately 80% without appropriate treatment. At this time, there is no accurate technique to predict this catastrophic complication in advance. In one study, radiomics was used on non-contrast brain CT scans within 24 hours after initial symptoms. The selected features could predict a malignant MCA infarction with an AUC of 0.91 [18].

Intracranial hemorrhage

Intracranial hemorrhage (ICH) is the second most common type of stroke, with a fatality rate of approximately 40% at one month and 54% at one year after bleeding. Only 12-39% of patients with ICH can achieve long-term functional independence [19]. The standard diagnostic technique for the detection of ICH is the non-contrast head CT. Differentiation between expansile versus non-expansile ICHs is crucial for patient management and decompressive craniotomy. Prediction of the early expansion of intraparenchymal hemorrhage on traditional imaging remains a challenge at this time. Radiomics has been used for this task using logistic regression models. Early promising results have shown that radiomics-based models can be more accurate than the current clinical-radiologic models. Adding new radiomics features to the clinical-radiologic models can improve the accuracy for predicting the chance of early expansion for each intraparenchymal hemorrhage on non-contrast CT [20]. Another similar study was performed involving over 1,153 patients with ICHs to predict hematoma expansion between the initial presenting head CT (within the first six hours of the admission) and the follow-up CT (performed after 72 hours). Three different prediction models were developed using the clinical and radiomics features (clinical model, radiomics model, and a hybrid model). The final assessment demonstrated that the hybrid model outperformed the other prediction models with an AUC of 0.820 [21]. Similar promising results have been reported for the radiomics technique in predicting the expansion of the hypertensive ICHs. In this pure radiomics analysis, a total of 576 features were extracted from the hypertensive ICHs on non-contrast CT. The subsequent LASSO model selected five features to be significantly associated with future expansion. The final model differentiated non-expansile from expansile hematoma with the sensitivity, specificity, and accuracy of 0.808, 0.835, and 0.820, respectively, on the test cohort [22]. In another similar study, the radiologist-based prediction model did a fair job in differentiating non-expansile from expansile parenchymal hematomas on non-contrast CT. The radiologist-based prediction model used the classical features for brain hematoma, including location, shape, density, hypodensities within hematoma, swirl sign, blend sign, black hole sign, and island sign with an AUC of 0.81. On the other hand, the radiomics pipeline developed with 1,942 extracted features, subsequent feature selection by the LASSO model, and logistic regression prediction had a higher AUC of 0.89 [23]. The ability of radiomics to predict hematoma expansion was evaluated in the other study involving 167 patients; 1,227 texture features were extracted from parenchymal hemorrhages on non-contrast CT. After feature selection, four features were found to be significantly associated with expansile parenchymal hematoma. A total of 23 different machine learning models were developed from these selected features. The best performance was with the linear support vector classifier with an AUC and accuracy of 72% [24].

Radiomics analysis has also been used to evaluate small intraparenchymal hemorrhages (less than 10 ccs). Adding the radiomics-extracted features from non-contrast CT to clinical features has significantly improved the model’s accuracy to predict poor outcomes in patients with small intraparenchymal hemorrhages with an AUC of 0.95 [25]. In another study, 129 patients from two different institutions with ICH were evaluated. The initial hematoma was manually segmented on non-contrast CT, and radiomics features were extracted from the segmented hematomas. The most important extracted features were selected using the LASSO model. Four radiomics features were found to be the most useful to predict expansile hematomas. Adding the selected features to the presence or absence of satellite lesion around a hematoma improved the performance of the logistic regression model to predict expansile hematomas with an AUC of 0.85, a sensitivity of 95%, and a specificity of 76% on the external validation cohort [26].

Neoplastic Versus Non-neoplastic Intracranial Hemorrhages

Differentiating between neoplastic and non-neoplastic brain parenchymal hemorrhages (e.g., hypertensive hemorrhage) is challenging in conventional radiology, especially on non-contrast head CT. In one study, the parenchymal hemorrhage and associated edema were segmented on non-contrast CT for radiomics evaluation. After feature extraction and feature selection, 100 most predictive radiologic features were selected to train random forest models. The subsequent trained model differentiated neoplastic and non-neoplastic intraparenchymal hemorrhages with an AUC of 0.89 using head CT images, significantly outperforming two radiologists [27].

Intracranial aneurysm

Intracranial aneurysms are frequently found in patients undergoing brain imaging for unrelated indications such as stroke, multiple sclerosis, headache. These aneurysms can have an inherent risk of rupturing, resulting in life-threatening subarachnoid hemorrhages and even death. These are typically followed by serial MRA or CTA to assess for interval change in size. Aneurysms vary in size and morphology and have a variable risk of rupture depending on multiple factors that are invisible to radiologists’ eyes [28]. Radiomics may have an application in the prediction of the chances of future rupture of these aneurysms. In one study on brain CTA, comparing the predictive models based on the morphology of aneurysm (radiologist diagnosis), models based on the morphology of aneurysm plus the “shape” radiomics features, and predictive models based on only multiple radiomics features, the last predictive model based on various radiomics features was significantly better than other models for prediction of aneurysm rupture with an AUC of 0.87 [29]. In one study on 719 aneurysms, 12 morphologic features were extracted from three-dimensional-digital subtraction angiography (3D-DSA) angiogram. The LASSO regression demonstrated that “flatness” is the most crucial predictive feature for aneurysm stability. The subsequent model based on the radiomics morphology and clinical feature predicted the aneurysm stability with an AUC of 0.85 [30]. As is evident, only morphologic radiomics features (a subset of radiomics features) were used to predict aneurysm stability. There are no data about the other types of radiomics features (e.g., texture analysis). Not all studies are promising; in one study on 3D-DSA, the radiomics morphology features (extracted by Pyradiomic) were inferior to traditional morphology analysis (done by MatLab software) for predicting aneurysm stability [31]. In the future, radiomics analysis of aneurysms may help physicians design a better follow-up strategy and reduce the patients’ radiation dose.

Demyelination

Inflammatory demyelinating disorders are a heterogeneous group of conditions characterized by acute or chronic inflammation involving the myelin, followed by reactive astrogliosis. There are different subtypes of demyelination such as multiple sclerosis (MS), neuromyelitis optica spectrum disorders (NMOSD), Marburg-type MS, concentric sclerosis of Balo, and acute disseminated encephalomyelitis [32].

Differentiating NMOSD and MS remains challenging in the clinic as well as on neuroimaging. Radiomics techniques have been implemented on T2-weighted sequences for this task. After extraction of 273 features, the LASSO-based logistic regression model selected 11 characteristic features with significant predictive capability. The final model based on these features and five clinical features (age, gender, antibodies to aquaporin-4, oligoclonal band, and spinal lesions) could differentiate NMOSD from MS with an AUC of 0.93 [33]. In another study, 485 radiomics features were extracted from spinal cord lesions. From the extracted features, nine were significantly different in MS and NMOSD. The final model, based on the nine extracted features, the size of the lesion, patients’ age, and expanded disability status scale, was able to differentiate multiple sclerosis from NMOSD with an AUC of 71.95 [34]. Radiomics has been used for the prediction of visual accuracy and outcomes in patients with optic neuritis. Seven radiomics features have been reported to be useful for predicting the patients’ visual accuracy and prognosis with the first episode of optic neuritis [35]. Estimating the age of removing plaques is also important in managing patients with demyelination and evaluating treatment response. Radiomics have been used for this task, and the features have been extracted from the T1, T2, fluid-attenuated inversion recovery, post-contrast T1, and quantitative susceptibility mapping sequences. The subsequent random forest models were able to estimate the age of the plaques with a median absolute error of 5.98 months [36].

Limitations

The common non-oncologic neurologic disorders studied by radiomics are summarized in Table 1. As shown in Table 1, the performance of radiomics analysis is promising in many different neurologic conditions. However, there are significant limitations as well as most of the publications are retrospective and based on small datasets from single medical centers. Moreover, there is significant heterogeneity in the radiomics pipeline design. There is no agreement regarding the radiomics software, the number of extracted features, the number of selected features, and the machine learning models. In addition, trained models are often not accessible to other researchers and many times not reproducible by other investigators. Such models end in publications and get cited, but unfortunately, have been unable to reach clinical implementation yet.

**Table 1:** Non-oncologic radiomics applications in neurology disorders. AI: artificial intelligence; NCCT: non-contrast computed tomography; NA: not applicable; SVM: support vector machines; DT: decision trees; AUC: area under the curve; IV: intravenous; CTA: computed tomography angiography; LASSO: least absolute shrinkage and selection operator; 2D: two-dimensional; 3D: three-dimensional; ANOVA: analysis of variance; DSA: digital subtraction angiography; MS: multiple sclerosis; NMOSD: Neuromyelitis optica spectrum disorder; STIR: short tau inversion recovery; QSM: quantitative susceptibility mapping; FLAIR: fluid-attenuated inversion recovery; RIA: radiomics image analysis

Reference number	Target	Imaging	Number of patients	Extracted features	Selected features	Software for feature extraction	Software for feature selection	AI model	Findings	Limitations
[13]	Detection of hyperacute infarction on non-contrast CT	NCCT	139	10	6	Run-length matrix	NA	SVM, DT, AdaBoost	AUC of 0.82 for the detection of hyperacute infarct. No difference between two and eight hours from symptom onset. The performance of the classifiers did not depend on the size of the infarction	No external validation group and the study considered the contralateral hemisphere as normal
[15]	Prediction of successful thrombectomy by radiomics analysis of thrombosis	NCCT	109 patients: retrospective training; 47 patients: prospective validation	1,485	9	Pyradiomics	Univariate feature selection	SVM	AUC of 0.88 to predict the successful first passage. AUC of 0.76 to predict the number of passages required for successful recanalization	Single-center study, the target was radiologic recanalization and not patients’ prognosis; manual segmentation
[16]	Prediction of recanalization after IV alteplase treatment from radiomics analysis of thrombosis	NCCT and CTA	67	326	38	MatLab	Linear discriminative analysis	SVM	AUC of 0.85 for prediction of recanalization after IV alteplase treatment using a combination of radiomics features of NCCT and CTA. The performance of radiomics was superior to traditional analysis of thrombosis (thrombosis length, thrombosis volume, etc.)	Small dataset. The results were reported by cross-validation, and there was no external validation cohort; manual segmentation
[17]	Detection of high-risk carotid atherosclerosis	T1, T2, T1+C, dynamic contrast-enhanced	162	788	33	ITK-SNAP	LASSO	LASSO	AUC of 0.989 in the training cohort and 0.986 in the test cohort for detection of high-risk plaques. Radiomics model and radiomics+ traditional model were better than traditional (human-based) model alone	Small dataset. No external validation. Manual segmentation. Radiomics analysis was based on single axial (2D) images at the largest plaque area, and 3D analysis was not performed
[18]	Prediction of malignant acute middle cerebral artery infarction	NCCT CTA	Train: 87 Test: 39	396	8	Artificial intelligence kit	LASSO	Multivariate logistic regression	AUC of 0.91 on test group to predict malignant infarcts	Retrospective. No clinical data were used. No external validation dataset
[20]	Prediction of the hematoma expansion	NCCT	Train: 182 Validation: 79	322	9	Artificial intelligence kit	LASSO + regression	Multivariate logistic regression	AUC of the clinical-radiologic model of 0.766. AUC of radiomics model for validation cohorts of 0.850. AUC of radiomics + radiologic model in validation cohorts of 0.867	Single-center retrospective study. No clinical data, just radiomics from hematoma. No external validation dataset
[21]	Prediction of the hematoma expansion	NCCT	Train: 864 Test: 389	396	3	Artificial intelligence kit	LASSO	Logistic regression	The radiomics model was better than the human-based model. Radiomics + human-based model was superior to each of model individually	Retrospective single-center study. No external validation
[22]	Prediction of the hematoma expansion	NCCT	Train: 149 Test: 105	576	5	Pyradiomics	LASSO	Regression analysis	Accuracy of 82% in the test group to differentiate expansible versus non-expansible hematomas	Single-center retrospective study. No external validation dataset
[23]	Prediction of the hematoma expansion	NCCT	Train: 177 Test: 74	1942	22	MatLab	LASSO	Univariate analysis and multivariable logistic analyses	Better performance of the radiomics model in comparison to the radiologist-based model	Single-center study. Only supratentorial hematomas were included. Retrospective study. No external validation. No clinical data were used. Manual segmentation
[24]	Prediction of hematoma expansion	NCCT	167	1,227	4	MatLab	Pearson correlation	23 different ML models	Best performance by linear SVM: accuracy of 72.6%	Single-center retrospective study. Radiomics was done on 2D images. Manual segmentation
[25]	Prediction of hematoma expansion	NCCT	313	396	58	Artificial intelligence kit	LASSO	Multivariate logistic regression	Addition of radiomics to clinical factors significantly improved the prediction of hematoma expansion	Single-center retrospective study. Only small hematomas <10 ccs were included. No external validation dataset
[26]	Prediction of hematoma expansion	NCCT	Train: 68 External validation: 61	396	4	Artificial intelligence kit	ANOVA-Kruskal-Wallis test and LASSO	Multivariate logistic regression	AUC of 0.85 in external validation. (This number appear realistic because it was tested on an external dataset of another medical center)	Retrospective study
[27]	Differentiation between neoplastic and non-neoplastic hematoma	NCCT	77	2,713	100	Pyradiomics	Gini impurity measures	Random forest	Radiomics and machine learning yielded equal or superior performance in comparison to radiologists	Small dataset. Single-center retrospective study
[29]	Prediction of aneurysm rupture	CTA	122	107	89	Pyradiomics	LASSO	Multivariate analysis	The radiomics model was better than the traditional (morphologic) model. The combination was better than each model individually	Small dataset. Retrospective single-center study. No clinical data were built to build the models. Aneurysms were followed for only 2 years
[30]	Prediction of aneurysm rupture	3D-DSA	420 (aneurysms)	12	4	Pyradiomics	LASSO	General linear, ridge, and LASSO	AUC of 0.85 to predict aneurysm rupture. No difference between the different models	Retrospective single-center study. Only aneurysm between 4 to 8 mm included. Most of the aneurysms ruptured
[31]	Prediction of aneurysm rupture	3D-DSA	353 aneurysms	13	13	Pyradiomics	NA	Univariate analysis	Traditional models were better than radiomics-based models	Retrospective single-center study
[33]	Differentiation between MS and NMOSD	T2 (3 T)	NMOSD: 77 MS: 73	273	11	Not mentioned	LASSO	Multivariable analysis	Model based on selected radiomics features + 5 clinical features: AUC of 0.93 to differentiate between MS and NMOD	Only T2 sequences. Optic nerves were not evaluated. Only 2D images were used
[34]	Differentiation between MS and NMOSD	T2	MS: 67 NMOSD: 68	485	9	Not mentioned	LASSO	Multivariable logistic regression analysis	The model was built on radiomics + clinical features: AUC of 0.88 in the training dataset. AUC of 0.71 for the prospective validation group	Only T2 images of the cord were used. Cross-sectional study and no follow-up was performed. Single-center study
[35]	Prediction of visual function on the first episode of optic neuritis	STIR and T1 fat sat+C	25	91	7	Pyradiomics	LASSO	Multivariate logistic regression	Radiomics may predict the visual outcome in optic neuritis	Small dataset
[36]	Estimation of the age of demyelination plaques	T1, T2, FLAIR, T1+C, QSM	32	44	NA	RIA R package	NA	Random forest	Estimation of plaque age with a median absolute error of 5.98 months	Different MR scanners. Small dataset

## Conclusions

Radiomics is an emerging research field in radiology based on the extraction of image information beyond obvious visible information. Although oncologic imaging is the most widely studied field, preliminary results regarding the applications of radiomics in non-oncologic neurological disorders are promising. Based on our current knowledge, radiomics solutions have been used for infarct detection on non-contrast brain CT, thrombosis characterization on CTA, identification of high-risk carotid plaque on MRI, prediction of malignant MCA infarction on CT, prediction of early expansion for cerebral hemorrhage on CT, differentiation of neoplastic versus non-neoplastic cerebral hemorrhages on CT, prediction of cerebral aneurysmal rupture on CTA, and characterization of demyelination lesions on MRI with acceptable performance. However, this field is still in its infancy and many challenges must be resolved before widespread clinical application. The main challenges are the lack of a uniform approach in radiomics pipeline design, small datasets, lack of external validation datasets, and the inability of investigators to reproduce published work in diverse research and clinical environments. Further randomized controlled studies are needed before widespread clinical applications of radiomics in neurological disorders.
